# Appendicitis—A Possible Cause—The Use of the Ligature—Is It Necessary?

**Published:** 1898-07

**Authors:** Wm. T. Oppenhimer

**Affiliations:** President of the City Board of Health, Richmond, Va.


For the Texas Medical Journal.

### Appendicitis—A Possible Cause—the Use of the Ligature—Is It Necessary?

BY WM. T. OPPENHIMER, M. D.,
President of the City Board of Health, Richmond, Va.

Read before the Richmond Academy of Medicine and Surgery, May 24, 1898.

The subject for the evening's discussion, as announced in the notices, was appendicitis. I do not wish to take in such a vast subject, only to confine myself to the cause, the results of inflammation and certain procedures for relief. I have often been twitted for pressing the theory that so many diseases were due to the accumulation of gas in the intestinal canal. Possibly 50 per cent. of all cases of sickness is due to some irregularity, imprudence or defect in digestion. The question is asked, Why do we hear more of appendicitis now than formerly? I would answer that the disease was not so well known, and that possibly as much existed then as now, but under different names, e. g., many cases formerly diagnosed as peritonitis were fulminant appendicitis. But, nevertheless, I claim the disease is more frequent now. Possibly the cause may lie in improper food. Bread is the most common food, and the common baking powder used has caused more and different varieties of indigestion than formerly, probably affecting the digestive juices. I bring this out, although I have no statistics to prove it, for I believe that appendicitis is nothing more than indigestion in the appendix. Authorities on the subject refer to the blood vessels, sex, etc., when naming the causes. The point I wish to make is that it is the result always of an accumulation of gas; never of plugging of the artery or sloughing. I believe that the capillaries are so numerous that even with blocking of the artery collateral circulation is soon established.

In every case of appendicitis, the patient is more or less dyspeptic. It may even be his first attack. The resulting gas accumulating in the cecum, the appendix becomes blown up and its orifice is blocked. In recurrent cases, the orifice may be more and more narrowed with each succeeding attack, until it is finally occluded, the circulation is cut off entirely if the distension is great, and sloughing results.

In forcing gas into the cecum, the appendix is more distended at its apex than elsewhere, and least at its orifice, because of the presence of circular muscular fibers. Constant pumping in of gas may result in partial closure only, and adhesions may form; but when there is complete closure, the fulminant variety is produced, and, going on, protective abcesses. This statement regarding closure in the fulminating form must be so, because where the appendix is filled with pus, if it were not entirely sealed there would be drainage into the cecum, and it would be recurrent. To attest my belief in it, I have operated for appendicitis without using the ligature. Of course, in the recurrent form, where the operation is done between the attacks, the ligature should always be applied. The danger from it is that it might not be applied near enough to the cecum, leaving pus which may result in septicemia, peritonitis, etc. In safe hands, the operation is less dangerous without than with the ligature.

The points I have stated are altogether different from those heretofore brought forward, and I would like for the gentlemen present to think of them.

Why do more men than women suffer from appendicitis? The reason given by an authority is that in the latter sex the appendicular circulation is reinforced by a branch from the ovarian artery. I contend that it is because the circular muscular fibers around the orifice of the appendix are stronger in the male, the tension is greater, and, therefore, closure is more likely. I do not deny that the circulation in women may supply more blood.

The points brought out have great bearing on the treatment, namely, food. Indigestion of all forms should have the closest attention, for the first seizure may bring on an attack of appendicitis.

